# Everyday tactics in local moral worlds: E-cigarette practices in a working-class area of the UK

**DOI:** 10.1016/j.socscimed.2016.10.012

**Published:** 2016-12

**Authors:** Frances Thirlway

**Affiliations:** Department of Anthropology, Durham University, Dawson Building, South Road, Durham, DH1 3LE, UK

**Keywords:** Smoking cessation, Health inequalities, Ethnography, Agency, Gender, Informal economy, Addiction

## Abstract

Research into e-cigarette use has largely focused on their health effects and efficacy for smoking cessation, with little attention given to their potential effect on health inequalities. Drawing on three years of ethnographic research between 2012 and 2015, I investigate the emerging e-cigarette practices of adult smokers and quitters in a working-class area of the UK. I first use de Certeau's notion of ‘tactics’ to describe the informal economy of local e-cigarette use. Low-priced products were purchased through personal networks and informal sources for financial reasons, but also as a solution to the moral problems of addiction and expenditure on the self, particularly for older smokers. E-cigarette practices were produced in local moral worlds where smoking and cessation had a complex status mediated through norms of age and gender. For younger men, smoking cessation conflicted with an ethic of working-class hedonism but e-cigarette use allowed cessation to be incorporated into male sociality. Continued addiction had moral implications which older men addressed by constructing e-cigarette use as functional rather than pleasurable, drawing on a narrative of family responsibility. The low priority which older women with a relational sense of identity gave to their own health led to a lower tolerance for e-cigarette unreliability. I draw on Kleinman's local moral worlds to make sense of these findings, arguing that smoking cessation can be a risk to moral identity in violating local norms of age and gender performance. I conclude that e-cigarettes did have some potential to overcome normative barriers to smoking cessation and therefore to reduce health inequalities, at least in relation to male smoking. Further research which attends to local meanings of cessation in relation to age and gender will establish whether e-cigarettes have similar potential elsewhere.

## Highlights

• Smokers and e-cigarette users bought cheap products for moral and financial reasons.
• Smoking cessation conflicted with an ethic of hedonism but e-cigarettes did not.
• Older men saw e-cigarette use as functional in a narrative of family responsibility.
• Older women gave their health low priority and found e-cigarettes too much trouble.
• E-cigarettes had potential to overcome some normative barriers to smoking cessation.

## 1 Introduction

Following the invention of electronic cigarettes (or ‘e-cigarettes’) in China in 2003 and their introduction into European and North American markets in 2006, sales increased dramatically from around 2010 (Fagerstrom et al., 2015). E-cigarettes were quickly identified as a technology with the potential to disrupt the tobacco and pharmaceutical industries, public health and tobacco (Stimson et al., 2014 p. 654). Whilst their long-term health effects have not been established, e-cigarettes are generally recognised as less harmful than tobacco smoking (McNeill et al., 2015, Nutt et al., 2014) and may provide an opportunity to reduce the disease burden from tobacco use significantly (Stimson et al., 2014 p. 655). There has however been controversy around their potential to ‘re-normalize’ and to act as a potential gateway to smoking, as well as their health effects and effectiveness as a smoking cessation tool (Rooke et al., 2015 p. 1).

At the time of writing, most e-cigarette users in the UK were trying either to stop or to reduce their smoking. They were most likely to use a rechargeable model with a reservoir or tank together with refill bottles of ‘e-liquid’ made up of propylene glycol and/or glycerine, plus flavours and usually nicotine (ASH, 2014). Slightly more women than men reported e-cigarette use, and slightly more people of higher rather than lower socio-economic status (SES), defined as occupying managerial, professional and intermediate, rather than in routine and manual jobs (West et al., 2016b). Ever-use of e-cigarettes by smokers was relatively high, but translation into continuing e-cigarette use and smoking cessation was much lower. The available evidence suggested that reasons included dissatisfaction with products and safety concerns (McNeill et al., 2015 pp. 55–56).

### 1.1 E-cigarettes and health inequalities

The extent to which low-SES smokers are successfully using e-cigarettes to quit smoking is a key issue in addressing health inequalities in materially rich countries such as the UK and US where there is a strong inverse social gradient in smoking. Different smoking rates by SES explain a significant proportion of health inequalities – for instance, approximately fifty per cent of the inequalities in lung cancer risk (Menvielle et al., 2009). People with low SES are more likely to take up smoking but despite being as likely to try to quit as other smokers, they are less likely to succeed (Barbeau et al., 2004, Giskes et al., 2006, Hiscock et al., 2012, Kotz and West, 2009, Sorensen et al., 2004). For those people who are unable to quit smoking, continuing use of nicotine in the form of the e-cigarette might be a solution. To date only one published study has considered views and experiences of e-cigarettes amongst lower SES smokers and ex-smokers (Rooke et al., 2015), and none have considered e-cigarette practices in a community setting, despite continued smoking in high-income countries becoming increasingly concentrated in ‘islands’ of disadvantage (Thompson et al., 2007) where complex pathways link individuals, places and smoking (Pearce et al., 2012). In this paper I draw on three years of ethnographic research on smoking and cessation in a working-class area to present some emerging findings regarding e-cigarette practices which may shed light on the potential of e-cigarettes to address health inequalities.

### 1.2 Tactics and moral worlds

I start by describing the local informal economy of e-cigarette buying and selling, using de Certeau's notion of ’tactics'. Tactics involve people with little or no power using, manipulating and diverting the spaces developed by the strategies of the powerful (de Certeau, 1984 p. 30), as in the shanty-towns of North East Brazil where Scheper-Hughes described ‘the everyday, oppositional practices of the poor’ (Scheper-Hughes, 1993 [1989] p. 472). Like the ‘weapons of the weak’ (Scott, 1985), tactics are a form of resistance operating as hidden transcripts rather than out in the open (Scott, 1990). A convincing account requires an appreciation of what those using tactics are trying to achieve; anthropologists have been accused of seeing resistance everywhere whilst providing little insight into the deeper motivations of the actors involved (Brown, 1996, Kleinman, 1996 p. 206; Ortner, 1995). I show how both e-cigarette users and smokers shopped around for best value, protecting their moral position by minimising expenditure on their ‘habit’, and argue that tactics and resistance more generally are bound up with the construction and maintenance of a moral identity in ‘a local moral context that influences the behaviour of its members’ (Kleinman, 2010 p. 375). I then consider addiction as a moral problem and how this translated in my study site into a division of e-cigarette users on age and gender lines. Finally I investigate some barriers to smoking cessation relating to the locally appropriate performance of age and gender, and consider the potential of the e-cigarette to remove these.

### 1.3 The study

North West Durham in the North East of England is an area of low hills and river valleys dotted with small towns and villages separated by rough pasture and moorland; it was extensively mined for coal from the mid-nineteenth to the mid-twentieth century, when local pits closed and colliery sites were reclaimed and landscaped. I characterise the area as working-class in the light of its industrial past and post-industrial decline. It has experienced long-term population loss; there has been little post-1914 immigration and the population is 98% white (Office for National Statistics, 2011). At the time of this study, a high percentage of people worked in routine and manual occupations, educational qualification levels were relatively low and there were high levels of ill health and worklessness (NOMIS, 2016). Older men I met had worked in the collieries; later on both men and women worked in local factories, most of which have since closed; male research participants who had jobs tended to work in building, landscaping, waste disposal, warehousing or driving, whilst women did low-paid part-time caring, cleaning or catering work. Smoking prevalence in England between 2012 and 2015 was around 20%, but since most research participants were or had been engaged in manual work, the smoking rates I found were closer to the England figure for manual occupations of 25%–30% in this period (West et al., 2016a).

Ethical approval for the research was obtained from the Durham University School of Health Ethics Committee and the Anthropology Department Ethics Committee. The researcher handed out an information sheet to research participants which explained that she was conducting a study of smoking. Research locations have been obscured and names and other details of participants changed in the text to protect anonymity. Ethnographic methods were used involving lengthy involvement of the researcher in the field sites, which included several villages and small towns within a ten mile radius. Over the course of more than three hundred visits, relationships were built up with research participants which continued over months or years. Data collection methods included life story interviews, discussions, conversations and participant observation in everyday settings and community venues such as social clubs and shops as well as on social media. Data emerged in the course of a lengthy and gradual dialogic exchange, enabling my interlocutors and myself to move beyond defensive repertoires around smoking and cessation to more nuanced and thoughtful discussion. As Cornwell has pointed out, most people give a ‘public account’ of health matters when questioned by a stranger which differs substantially from the ‘private account’ they give to the same person after they have got to know them (Cornwell, 1984 pp. 11–17).

E-cigarettes were observed in the course of seventy-five field visits and discussed with forty-one participants with a mean age of forty-two and a range of eighteen to seventy-five, of whom twenty-eight were men and thirteen were women. Data were collected mainly in the form of hand-written field notes made either during or immediately after each visit and typed up the same day. Field notes included a detailed account of each visit including observations as well as conversations with research participants, reproduced as exactly as possible with some elements taken down verbatim. Quotes in this paper are taken from these notes. I undertook what Coffey calls ‘passionate analysis’ or an imaginative and creative engagement with the data (Coffey, 1999 pp. 136), organising narratives in different ways and investigating different explanatory models from the social sciences. I tabulated variables such as smoking and e-cigarette histories, preferred places of purchase, brands, models and flavours, and tested my analysis at various stages by searching and sorting field notes by particular themes using key words. I examined how individuals made sense of their own and wider histories and looked for local structures of feeling (Williams, 2003 [1977]).

## 2 Findings

### 2.1 Everyday tactics in the informal economy

Adam (30), tall and personable with a cheeky grin, was nursing a pint when I met him in a social club on a sunny Friday afternoon in 2012. He was working on a building site and had stopped in for a drink on his way home. At that time, Adam smoked illicit rolling tobacco at work and ready-made cigarettes at the weekend. I continued to see him now and again, and in 2013 I found he had switched to e-cigarettes, although he still smoked an occasional cigarette as well: *‘A lad at work brought one in, I tried it, liked it so asked him to get me one’*, he told me. In 2014 we had a longer conversation as we walked behind the village banner at the Durham Miners' Gala: ‘*You're going to say it's bad for us',* he said as he took a silver tank-style model out of his pocket, pressed the LED button to turn it on and invited me to have a puff. He laughed when I coughed as the tobacco taste hit the back of my throat. I asked him who made it but he didn't know: *‘It's the liquid that matters'*, he said. He showed me the refill bottle he was carrying in his pocket – a friend had bought it for him at a car boot sale.

Adam's description of buying e-cigarettes and e-liquids at informal outlets and through personal networks was typical of the local e-cigarette economy, which operated at an informal, unofficial level as a ‘world underneath’ (Castells and Portes, 1989). Research participants bought e-cigarettes and e-liquids at street markets, discount shops and car boot sales. On the high street of one small town were branches of two national pharmacy chains which had arrangements with large e-cigarette companies (including one owned by a tobacco company) to stock their products exclusively, but cheaper products were readily available in other high street shops including discount household goods shops, newsagents and hardware shops. The street also boasted a small open-air market where, in 2013, two stalls sold lighters, filter tips and papers (for smoking tobacco), grinders, bongs and blunt wraps (for cannabis use) as well as basic tank e-cigarettes and refill bottles in five or six flavours. By 2014, a small specialist e-cigarette stall was in place, displaying half a dozen tank models alongside a rainbow array of refill liquids in more than fifty flavours and strengths, with multi-buy offers prominently displayed. Barry (55), well-wrapped up in a down jacket and beanie hat, with a large silver tank e-cigarette hanging from a lanyard round his neck, told me the liquids were made in China and repackaged for him - with a prominent Union Jack logo and local place name, I noticed. By 2015, Glenn, a younger man using a tank model with a mouth piece shaped like a skull, had replaced Barry: *‘He went out of business’*, Glenn told me: ‘*trading standards were after him for the dodgy liquids’*.

De Certeau's tactics are continually mobile; they ‘poach’ and ‘create surprises’ within the spaces of power (Round et al., 2008 quoting de Certeau, 1984 p. 38). Buyers and sellers in the local informal e-cigarette economy were constantly on the move: entrepreneurs set themselves up in business then disappeared as others took their place; consumers shopped around, constantly adjusting their buying practices to gain a small price advantage, as another stallholder told me ruefully in June 2015: *‘Our customers are going to the other stall because it's cheaper, but our flavours taste better’*, he complained. Better value was cited by research participants as one reason for their overwhelming preference for tank-style models and refill bottles over the disposable ‘cigalikes’ and replaceable cartridge models, which worked out far more expensive per puff. For the very poorest smokers, cost was a barrier to any e-cigarette use; when I met Martin, unemployed and volunteering at a local community project, he was smoking thin ‘rollies’ as he worked. I asked if he had tried e-cigarettes, but they were ‘*too expensive*’, he told me. Although £10 would buy a starter tank and e-liquid, smokers like Martin could get a week's worth of illicit rolling tobacco for the same money and could not risk such a large outlay on something that might not ‘work’ for him.

Smokers as well as e-cigarette users switched brands and products to get the best deal, mixing and matching economy cigarettes, rolling tobacco and illicit tobacco - manufactured illegally or on which tax had not been paid - in line with their fluctuating finances over the week or month (Gilmore et al., 2014, Stead et al., 2013 p. 2214). One village shopkeeper told me how most of his clientele bought the cheapest economy cigarette brand available, then switched to another brand if it took over the cheapest market position. The lack of a clear division between consumers and suppliers in the e-cigarette economy was replicated both in the illicit tobacco market and in the informal economy more generally, as people bought from their friends or became entrepreneurs themselves if the opportunity arose. In 2012 when scented espadrilles were fashionable locally, research participants took advantage of cheaper prices in Spain to bring multiple pairs back from holiday and sell them on. One young man had hundreds of pairs in the back of his car and sold them round the villages.

Whilst the local market for both tobacco and e-cigarettes shared many of the characteristics of the informal economy more generally, buying cheap tobacco and e-cigarettes was not just about getting value for money. Older users – even those who were financially secure - were particularly likely to limit their expenditure on their e-cigarettes: Ian (50), who started out in factory work but now headed a community project, shopped around and bought cheap: *‘I got the battery and the liquids I think from a stall on the market, I did get the same battery from the other stall but it came apart, I got my money back. The one from this stall was £10 but it lasts for three days’,* he said. Barbara (60), whom I met by Barry's stall in 2014, saved money when she was still a smoker by buying illicit cigarettes locally as well as bringing back duty-free tobacco from her frequent holidays abroad. When I met her, she had switched to an e-cigarette some months before and was considering upgrading to a £20 e-cigarette with a bigger battery; Barry showed her a £40 model but she said this was too much to pay. Many studies have shown that illicit tobacco and the informal economy are not seen as a moral problem in low-SES communities (Gough et al., 2013, L' Hoiry, 2013, Stead et al., 2013, Wiltshire et al., 2001); I suggest in the following sections that the sourcing of cheap tobacco and e-cigarettes was in fact a solution to the moral problems of addiction and expenditure on the self, particularly for older smokers.

### 2.2 Negotiating addiction and the moral meanings of e-cigarettes

The question as to whether e-cigarette use was *morally* better than smoking – as opposed to healthier - was spontaneously raised by a number of e-cigarette users and potential users, for whom addiction of any kind clearly represented a moral problem. As many have argued, addiction represents deviant desire and is commonly seen as fulfilling some psychological deficiency or providing a dysfunctional way of coping (Bell and Keane, 2012, Keane, 2002a, Keane, 2002b, Quintero and Nichter, 1996, Room, 2003). Because of the common element of addiction, some research participants explicitly equated smoking and e-cigarette use in moral terms, and declined to switch to e-cigarettes for this reason. Craig (18) argued: *‘One of my mates was on them for three weeks, then his battery ran out and he was fidgeting like mad - you're still addicted, it's not better’*. When I saw Craig's mother a year later, she told me proudly that he had stopped smoking in order to pass soldier selection fitness tests and join the British Army. I was not surprised to hear, given his views about e-cigarettes, that Craig had used pharmacotherapy (NRT or varenicline) to quit. Others did move from smoking to e-cigarettes, but then felt they should give up the e-cigarette as well: although Ian had stopped smoking and used an e-cigarette for eighteen months when I spoke to him, he struggled with the moral implications of long-term use: *‘Now I need to start getting off the e-cigarette as well’,* he said. I asked why: ‘S*o I can be off the nicotine altogether’,* he told me.

Faced with continued addiction to nicotine as a moral problem, some e-cigarette users sought to protect their moral position by positioning the e-cigarette as a functional cessation aid, thus establishing their moral legitimacy as reformed smokers. The association of the e-cigarette with pleasure or ‘play’ was resisted; older research participants were particularly likely to avoid aspects of e-cigarette use which smacked of frivolity or self-indulgence: sweet flavours, expensive parts, vaping culture and language. Ian told me: ‘*I use the eighteen milligram strength, tobacco flavour, I can't see the point of the other flavours – I've tried ‘em, like had a puff off someone but it was horrible’*. Cleaner Margaret (70) added: *‘If it doesn't taste like a cigarette then what's the point, how could it replace it?’* Older smokers such as Margaret were particularly likely to combine a suspicion of the new with an element of class hostility to an artefact seen as ‘pretentious’ (Bennett et al., 2009 p. 109).

Contrasting ideas of e-cigarette use as functional or recreational were reproduced in commercial settings, which also illustrated how e-cigarettes carried different meanings according to age and gender. In one shop with a funky graffiti-style black and purple frontage and a ‘vape’ name, the owner Neil and a couple of customers, all men in their thirties with carefully-styled beards and intricate sleeve tattoos, were sampling a toffee donut flavour and blowing out huge clouds of vapour. One of them told me he regularly travelled to the shop from another town twelve miles away, and had spent hundreds of pounds on different e-cigarette components and flavours. Neil and his customers were similar to the ‘vapers’ often featured in lifestyle journalism (Burn-Callander, 2015, Haynes, 2015, Shanahan, 2015): focused on the e-cigarette as pleasure rather than cessation aid and willing to experiment and spend out to get the best flavour and vapour, with some preferring no nicotine as it *‘spoiled the flavour’*. This hobbyist demographic of largely younger men with relatively high incomes was, with the exceptions I have just described, largely absent from my field sites. More typical was a tiny old man leaning on a walking stick, who came into Neil's shop while I was there. *‘He's eighty-four’*, Neil told me after serving him; *‘He kept coming in saying it was “going wrong”; he couldn't really work it, in the end I just gave him a more basic model’*.

The functional/recreational division was reproduced on a smaller scale on Glenn's market stall. Although not much more than a metre wide, it was divided into two clear sections: on the left stood a large inclined wooden tray of the usual refill bottles of 10 ml in their rainbow colours and flavours: *‘They're Chinese’,* Glenn said dismissively, *‘they have propylene glycol in’.* On the right were his favourites, an array of 30 ml bottles in plastic shrink wrap and poorly-printed labels, with a ‘vape’ name. This British brand, he told me, used vegetable glycerine instead: *‘It makes the big vapour clouds, the cloud chasers’*, he said proudly. In another market town, one of the two e-cigarette shops on the depressed high street was similar to Neil's, with a vape name and a funky animal logo, whilst the other had a white, blue and green colour scheme recalling UK health (white and blue) and pharmacy (green) settings, and a name referring to health and smoking. The assistant in this second shop described her customers as: *‘older ladies - they come in here, rather than going to the one further up, because there's lots of young lads there - I think with me being female, they prefer it.’* For these customers, male-dominated recreational vaping culture appeared to be a barrier; in fact Neil and his customers were the only people I met who used the term ‘vape’. As in a recent study (Rooke et al., 2015), most research participants were somewhat at a loss as to terminology; Lisa (37), unemployed and living in run-down private rented accommodation, told me she smoked, but that *‘a lot of my friends have started using them new things …* ’ She hesitated, looking for the word; *‘You mean e-cigarettes?’* I said. That was it, she agreed; she had tried *‘the ones that look like cigarettes’* but they gave her mouth ulcers, though these are in fact a common side-effect of smoking cessation (McRobbie et al., 2004); *‘I haven't tried the ones with the liquid though, the ones my friends have’.* Many people referred to *‘them electronic ones’* or *‘electric ones’*; Barbara spoke of her *‘pipe’.* This lack of agreement about terminology was symptomatic of the different meanings attached to e-cigarettes by different users.

### 2.3 Gendered moral worlds of smoking and cessation

Whilst some research participants construed addiction as a moral problem, this was not the only normative issue at stake. Smoking and cessation had a complex moral status mediated through other local norms including the appropriate performance of age and gender (Broom and Warin, 2011, Butler, 1990; Courtenay, 2000, Saltonstall, 1993), and e-cigarette use inevitably became enmeshed in these broader issues. Few studies of smoking have attended explicitly to normative considerations which might conflict with cessation; the moral imperative of health (Lupton, 1995) is so well-established that the possibility of conflicting norms is rarely even admitted. Notable exceptions include Robinson and colleagues' work on the moral difficulties of UK mothers trying to establish smoke-free homes (Phillips et al., 2007, Robinson, 2008, Robinson and Holdsworth, 2013, Robinson and Kirkcaldy, 2007) and Bottorff and colleagues in Canada on how parents of young children negotiate their smoking (Bottorff et al., 2006, Greaves et al., 2010; related articles from 2000 to 2010). Similar issues have been considered in relation to food practices and obesity (Stead et al., 2011, Warin et al., 2008). These studies all used a design which enabled researchers to attend to relational and in most cases familial aspects of participants' lives.

One area in which relational and familial matters came to the fore was in research participants' moral identities. For some women, smoking cessation - but also e-cigarette use for reasons I will explain - appeared to fail largely because looking after their own health came relatively low down their priority list. Kim (46), a cleaner who worked all hours to put her son through university, smoked thin cigarettes she rolled herself, except on nights out when she opted for ready-made cigarettes. Kim was a serial quitter and had tried everything to give up; she bought an e-cigarette in 2013 and this went well until *‘it rolled off the table and broke’* and she reverted to the pouch of illicit rolling tobacco tucked into the front pocket of her tabard. Retired home carer June (65) smoked economy cigarettes; she was generally busy all day cleaning and shopping for her elderly father, taking her brother to hospital appointments or giving her grandchildren tea. *‘She's the family social worker’*, her father liked to say. Kim and June were typical of mature adults and especially women in my study in having what Warin has described as a relational sense of identity in which care of the self was at the bottom of a hierarchy of concerns (Graham, 1984; Warin et al., 2008 p. 104). For these women, their own health simply did not seem important enough, in comparison with the many tasks they did for others, for them to put in the effort needed to quit. As a result, women like Kim and June, whilst they might try an e-cigarette, quickly became impatient with aspects in which it compared poorly with the ever-reliable, endlessly replaceable cigarette. This issue links to my earlier point about the limited financial investment which older research participants were willing to put into either tobacco or e-cigarettes; the expenditure of significant time or money on the self was not compatible with moral identity as a caring adult invested primarily in nurturing relationships with others.

Over the course of the study I saw research participants struggle with the time, effort and expense involved in finding the ‘right’ e-cigarette and the frequency of product failure i.e. cheaper tank models splitting, leaking, or bubbling if over-tightened or dropped, and problems with batteries running out or failing to charge; unless users were highly motivated to quit, smoking was significantly easier, and often cheaper taking into account the cost of e-cigarette replacement and the ready availability of illicit tobacco. Participants in a recent study described how e-cigarette use required ‘vigilance and planning’:

‘The e-cigarette devices would need constant monitoring. For example, the users needed to make sure, before they left home, that they had enough cartridges or e-liquid with them and the battery was fully charged. If in the middle of a party or a concert the battery died or they ran out of cartridges or e-juice, finding another e-cigarette was not as easy as finding another cigarette’ (Pokhrel et al., 2015 p. 4)

Another area in which smoking cessation conflicted with local norms was in relation to an ethic of working-class hedonism which not only encouraged sociable smoking and drinking, particularly among younger men, but also positioned abstention or cessation as morally problematic in terms of either separating oneself off and being pretentious or ‘snobby’ (Bennett et al., 2009 p. 209) or being insufficiently masculine (O'Brien et al., 2009). Sociality could be enforced: several people described being bullied into smoking relapse during the course of a night out with friends. Enjoyment could even be a matter of pride; builder Malcolm (47), a heavy smoker and drinker, told me he did not envy the middle-class inhabitants of the area where I lived, despite our longer life expectancy: ‘*Them at Greendale haven't enjoyed themselves the way us lot have - I've got no regrets',* he told me. In a characterisation which recalled Willis's classic account of ‘lads’ and ‘ear ‘oles’ (Willis, 1977), smoker Shelley (23) explained the pecking order which prevailed at school when she was fourteen: *‘There were the swots who didn't drink or smoke, the Goths and Emos who smoked dope, and the popular kids - I was in that group - we all smoked’*.

This ethic of hedonism applied particularly to younger men and could make it difficult for them to give up smoking. However e-cigarette use had the potential to take on an association with hedonistic masculinity as we saw with Neil and his customers - not least because an interest in gadgets and technology was already a legitimate male trait, thus making the e-cigarette a doubly viable masculine accessory in combining hedonism with technology. Although I met no hobbyist vapers other than Neil and his customers, there was some evidence that this was beginning to change locally; when I last saw Adam, he was very proud of his latest, fourth-generation e-cigarette with wireless connectivity, and he told me that several of his friends had followed his example.

Local masculinity was performed differently in mature adulthood (Evans et al., 2011), at which point smoking cessation could function as part of a narrative of family responsibility (Bottorff et al., 2009) or indeed of mastery (Hanninen and Koski-Jannes, 1999) just as long as no weakness was shown; it was important that smoking cessation by men should be instant – a triumph of the masculine will (White and Baird, 2013). Retrospective narratives of cessation described an instant decision to quit, usually without help. Some of the expressions men used were: ‘*I just stopped … threw the pack in the fire … threw down the pack … never had another one’* – but several later confided that they had made earlier, unsuccessful attempts which were excised from the heroic narrative. Normative masculinity also required that one should give up smoking if suffering serious ill-health such as cancer, stroke or heart attack; not to do so in such circumstances implied weakness or a lack of self-control. In this context, e-cigarettes were sometimes deployed defensively by dual users of tobacco and e-cigarettes as a badge of moral intent or a sign of good faith in relation to pressure from relatives or doctors to quit smoking. Volunteer Simon (40) had a heart attack in 2014 and promised his family he would give up smoking; he told me he had tried before but had a *‘bad reaction’* to the medication. He bought an e-cigarette but continued to smoke. Former glazier Mark (51) had multiple chronic health conditions including spondylitis and diabetes. He was using an e-cigarette when I first met him in 2013, but a plastic box containing filter tips, tobacco pouch and cigarette papers sat on the counter at the project where he volunteered, and he was often out on the pavement smoking. In November 2014, when I saw Mark helping to unload a van despite his bad back, he assured me that the recessed filter of the (illicit) cigarettes he was smoking made them safer. In any case, he told me, he was going to the market that week to buy a new e-cigarette. Mark was still smoking when I last saw him in 2015. I am not suggesting that Simon or Mark acted cynically; I interpreted their assurances that they were taking active steps to address their smoking, for instance by using an e-cigarette or smoking a ‘safer’ cigarette, as designed to avoid loss of face and demonstrate that they were still in control. I generally had to get to know male research participants quite well before they were willing to admit that they struggled to quit smoking.

## 3 Discussion

Smoking and cessation in my study sites had a complex moral status mediated through other local norms including the appropriate performance of age and gender. One practical barrier to a successful switch from tobacco to e-cigarettes was the lengthy process of trial and error involved in finding an e-cigarette that was reasonably effective and reliable. Whilst this was an issue which potentially affected all users, the financial implications of multiple exploratory purchases were a greater concern for poorer smokers. There may be scope for improving product satisfaction and quitting success through the provision of peer support or expert user schemes such as have been organised in the UK by Leicester Stop Smoking Service (personal communication from Louise Ross, Leicester City Council, 9 September 2016); help to buy a ‘starter’ e-cigarette would address financial barriers. This practical problem was also a gendered issue inasmuch as the energy, time and money needed to switch successfully to e-cigarettes were often lacking for older women heavily invested in family care, whose personal health was relatively low in their hierarchy of concerns (Graham, 1984, Warin et al., 2008). There may be ways of ensuring that smoking cessation initiatives support the relational identity of some female smokers rather than undermining it, perhaps through family-centred interventions (Johnston and Thomas, 2008).

Although this could be a function of my study design i.e. how and where I met research participants, I found more men than women who were current e-cigarette users, despite broadly similar local smoking rates. Latest UK data showed slightly higher rates of e-cigarette use amongst women (West et al., 2016b), but this figure cannot tell us whether men and women e-cigarette users are equally likely to quit smoking. Women are more likely to use disposable or first-generation cigalikes (Dawkins et al., 2013, Piñeiro et al., 2016), but since second-generation devices deliver nicotine more effectively (Farsalinos et al., 2014) and are more satisfying to users (Dawkins et al., 2015), there is a risk that using less effective models may translate into fewer women ceasing to smoke, even if figures show slightly more women than men using e-cigarettes overall. There is no mention of gender in the most recent UK e-cigarette evidence review (McNeill et al., 2015); further research will be needed to establish whether there is a gender differential in the use of e-cigarettes to quit smoking successfully.

Turning now to men, smoking cessation in my field sites presented two immediate problems for locally normative masculinity. The first was that smoking cessation by younger men was seen as a rejection of the hedonistic values of male sociality. The second was that addiction was equated with weakness, and to try to stop smoking and then fail was a significant risk to moral identity. The advantages of e-cigarette use were that younger men could construct e-cigarette use as recreational (thereby denying addiction), whereas older men could position e-cigarette use as functional, as part of a narrative of smoking cessation linked to male familial responsibility. Some men with serious health problems who were unable to quit smoking remained dual users of tobacco and e-cigarettes, but deployed e-cigarettes as a badge of moral intent. Research participants thus discovered for themselves ways of using e-cigarettes which enhanced rather than detracted from local constructions of masculinity - an approach which has been used successfully in relation to other areas of health (Hunt et al., 2014a, Hunt et al., 2014b).

Returning to the issue of tactics, I described how research participants bought and sold e-cigarettes, tobacco and other goods mainly through informal outlets and personal networks which are not easily captured by official figures. Although some neighbourhood-level research has taken place (Hsu et al., 2013, Rose et al., 2014), studies of the e-cigarette market have focused on larger suppliers engaged in formal marketing, particularly via the internet (Bauld et al., 2014, De Andrade et al., 2013, Zhu et al., 2014); more research is needed on the informal economy where low-SES smokers are concentrated. For older users in particular, sourcing cheap e-cigarettes (or indeed tobacco) involved saving money, but also recovering agency, choosing to spend less and thereby also demonstrating moral worth in relation to the moral problems of addiction and expenditure on the self. This brings de Certeau's notion of everyday tactics into play, with the object of such tactics being to attain and hold onto self-respect or moral worth. Relating this to anthropological critiques of resistance more generally, I argue that rather than *itself* having moral content, resistance becomes necessary in the negotiation of local moral worlds. De Certeau recognised this; indeed he has been criticised for regarding tactics as inherently morally good (Mitchell, 2007). What is key to his thinking is rather a preoccupation with the individual moral project, by which I mean that tactics, for de Certeau, are always bound up with the construction and maintenance of a moral identity.

This account of the interplay of e-cigarette use with local norms is not exhaustive but presents a snapshot of some of the identity work taking place in relation to e-cigarettes in one small area at a particular time. Some narratives are missing, most notably those of younger women as this was a demographic to which I had little access. Young men were most likely to buy into recreational vaping culture whereas older people saw e-cigarettes as more functional; older women with heavy family responsibilities quickly became impatient with product unreliability. I have explained how these trends reflected local age and gender roles in relation to smoking cessation more generally: how giving up smoking was a threat to male sociality; how struggling to quit was unmanly; and how cessation was a low priority for women invested in an identity of care. New norms relating to e-cigarette use evolved in the context of local meanings of smoking cessation; the success of the e-cigarette in addressing health inequalities will partly depend on whether it enables users to overcome these normative barriers to cessation. My findings suggest that it does have some potential to do this, at least in relation to men. However, the e-cigarette is constantly changing; should it become a more medicalized product, it might lose its attractiveness as a masculine accessory. On the other hand, if more reliable and effective models start to dominate the market, this will diminish the trial and error process which discourages older women users. Meanings can also change, and users are endlessly resourceful in finding ways of using products which chime with rather than detract from local constructions of age and gender.

The study was carried out in a specific place and findings cannot be generalised to low-SES areas across the UK or further afield; it is likely that a study in an urban environment would find that gender roles for instance were less strongly differentiated or operated differently. Nevertheless this study can give us some clues to e-cigarettes' potential to reduce the social gradient in smoking more generally. Obvious barriers likely to apply to low-SES areas beyond the study population include the cost of the first e-cigarette and liquid compared to illicit tobacco, as well as the subsequent expense involved in finding a reliable and effective model. Going beyond financial considerations, I suggest that local meanings of smoking and cessation in relation to gender (Amos et al., 2012; Bottorff et al., 2014) and age are crucial to e-cigarette take-up, as are place-based smoking and cessation practices more generally (Pearce et al., 2012). In this context, anthropology can play a key role in conducting in-depth place-based research and examining local culture as a dynamic influence (Goldade et al., 2012, Nichter et al., 2009). Attending to local moral worlds enables us to recognise that local norms relating to the performance of age and gender may make it risky, even dangerous to wellbeing to adopt health behaviours which might appear self-evidently positive (McKie et al., 2003, Stead et al., 2011, Warin et al., 2008), so that giving up tobacco or taking up e-cigarettes can be more of a risk to moral identity than carrying on smoking. Such a realisation will contribute to public health approaches which restore agency to smokers and e-cigarette users, recognising them as active producers of meaning.
